# Collaborating with AI in literature search—An important frontier

**DOI:** 10.1097/HC9.0000000000000336

**Published:** 2023-12-07

**Authors:** Masaru Enomoto, Cheng-Hao Tseng, Yao-Chun Hsu, Le Thi Thanh Thuy, Mindie H. Nguyen

**Affiliations:** 1 Department of Hepatology, Graduate School of Medicine, Osaka Metropolitan University, Osaka, Japan; 2 Division of Gastroenterology and Hepatology, E-Da Hospital, Kaohsiung, Taiwan; 3 Division of Gastroenterology and Hepatology, Stanford University Medical Center, Palo Alto, California, USA; 4 Department of Epidemiology and Population Health, Stanford University Medical Center, Palo Alto, California, USA

To the editor,

The growth of large language models in artificial intelligence (AI), exemplified by OpenAI’s Generative Pre-trained Transformers, is remarkable^1^ and can potentially aid in the systematic review of the ever-expanding medical literature. In fact, professional society guidelines are generally based on such data, but they are labor intensive.

In a 2020 Lancet Gastroenterology and Hepatology issue, we published a meta-analysis regarding HCC incidence with tenofovir disoproxil fumarate (TDF) versus entecavir in chronic hepatitis B.^2^ We spent hundreds of hours to screen >5000 studies to identify 31 studies eligible for further data extraction and synthesis^2^ (briefly, studies published between January 2006 and April 2020 with time-to-event data for incident HCC in TDF-treated or entecavir-treated patients).

Simulating the literature search for this study,^2^ we asked Generative Pre-trained Transformer-4, “Please find relevant studies with time-to-event data for incident HCC occurring in patients with chronic hepatitis B who received TDF or entecavir to know whether TDF and entecavir differ in their association with HCC risk in chronic hepatitis B patients.” Generative Pre-trained Transformers listed the titles, authors, journal names, and even PMIDs of “likely” papers that were mere hallucinations (Supplemental Table, http://links.lww.com/HC9/A698). Next, we asked the same question to another AI-generated research assistant, “Elicit.”^3^ After we clicked “show more” several times, in 10 minutes, Elicit showed 70 papers, with 48 of these published during the study period and 22 thereafter. At the end, of the 48 studies from the Elicit search that were published during the study period, 11 papers overlapped with those found by the traditional search, and 37 were newly identified (Supplemental Figure, http://links.lww.com/HC9/A699). In other words, 20 from the traditional search were missed by Elicit, and 37 were gained. Among the 20 missed papers, 5 were meeting abstracts, and 8 were published before 2018 and included only entecavir patients without a comparative arm. Thus, although 50,027/119,053 (42.0%) patients in the meta-analysis were missed in the Elicit search, only 1 (596 patients) out of 9 comparative studies was missed.^4^ Of the 37 additional papers “gained,” only 1 was an original research article (303 patients)^5^, which would have met our study criteria.

In summary, despite errors and miss rates with the current platform, systematic literature search using AI appears very promising, eliminating hours of human labor while improving search quality. As AI technology continuously evolves, efforts to refine and improve AI-based literature search platforms should be continued.

## Supplementary Material
