# Supplementary figures and images for: Collaborating with AI in literature search—An important frontier
Source: Hepatol Commun. 2023 Dec 7;7(12):e0336. doi: 10.1097/HC9.0000000000000336 (PMC10984654; doi:10.1097/HC9.0000000000000336)

## Slide 1
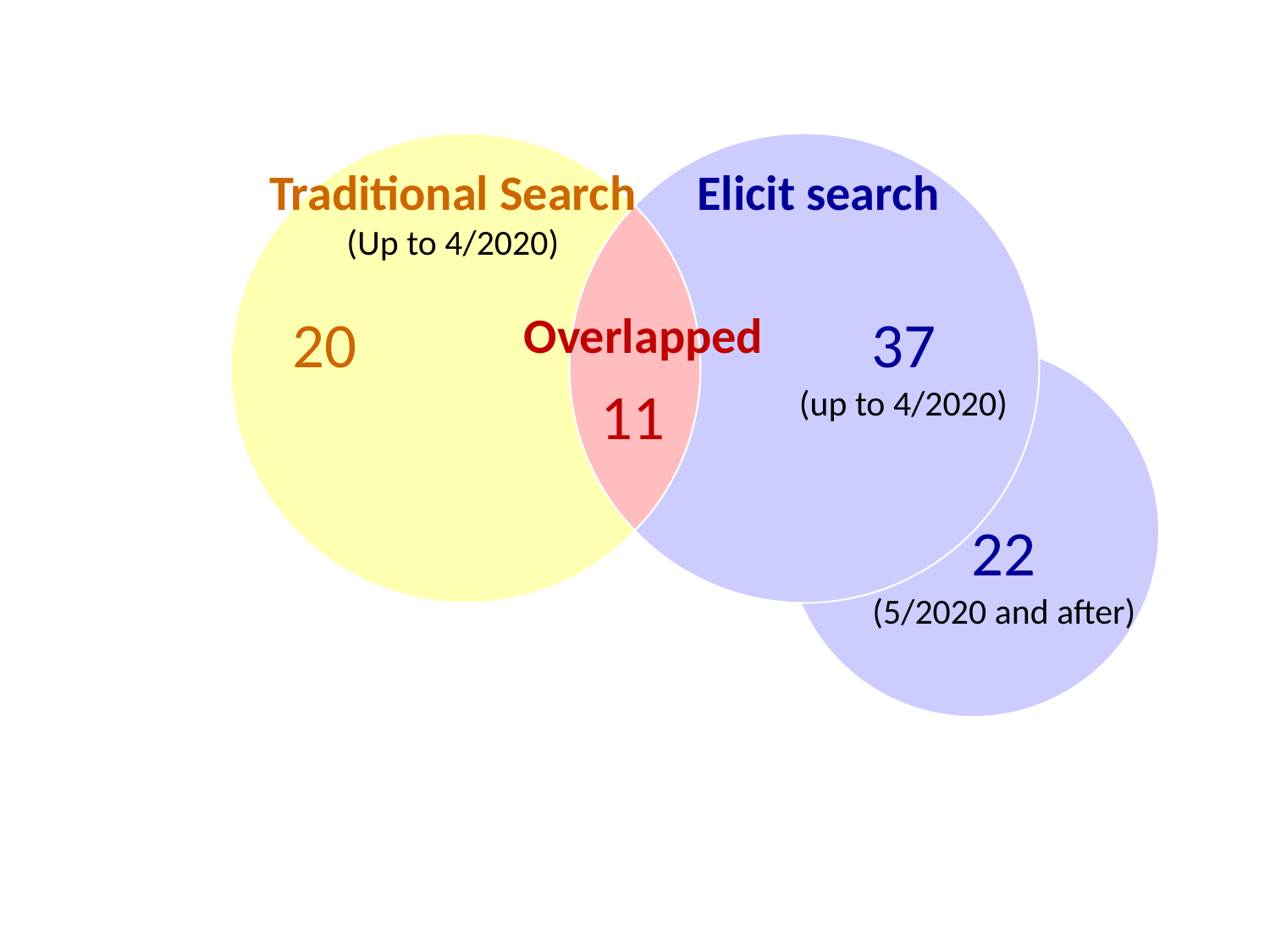

Traditional Search
(Up to 4/2020)
Elicit search
20
Overlapped
37
(up to 4/2020)
11
22
(5/2020 and after)

Supplement: Supplementary file 2 [file hc9-7-e0336-s002.pptx]
